# Bioinformatics Approach Predicts Candidate Targets for SARS-CoV-2 Infections to COPD Patients

**DOI:** 10.1155/2022/1806427

**Published:** 2022-06-21

**Authors:** Li Che, Guangshu Chen, Xingdong Cai, Zhefan Xie, Tingting Xia, Wei Zhang, Shengming Liu

**Affiliations:** ^1^Department of Pulmonary and Critical Care Medicine, Jinan University First Affiliated Hospital, Guangzhou 510630, China; ^2^Department of Endocrinology, Guangzhou Red Cross Hospital, Jinan University, Guangzhou 510220, China

## Abstract

COVID-19 is still prevalent in more world regions and poses a severe threat to human health due to its high pathogenicity. The incidence of COPD patients is gradually increasing, especially in patients over 45 years old. COPD patients are susceptible to COVID-19 due to the specific lung receptor ACE2 of SARS-CoV-2. We attempt to reveal the genetic basis by analyzing the expression of common DEGs of the two diseases through bioinformatics approaches and find potential therapeutic agents based on the target genes. Thus, we search the GEO database for COVID-19 and COPD transcriptomic gene expression. We also study the enrichment of signaling regulatory pathways and hub genes for potential therapeutic treatments. There are 34 common DEGs in the two datasets. The signaling pathways are mainly enriched in intercellular junctions between virus and cytokine regulation. In the PPI network of common DEGs, we extract 5 hub genes. We find that artesunate CTD 00001840, dexverapamil MCF7 UP, and STOCK1N-35696 PC3 DOWN could be therapeutic agents for both diseases. We also analyze the regulatory network of differential genes with transcription factors and miRNAs. Therefore, we conclude that artesunate CTD 00001840, dexverapamil MCF7 UP, and STOCK1N-35696 PC3 DOWN can be therapeutic candidates in COPD combined with COVID-19.

## 1. Introduction

Severe acute respiratory syndrome coronavirus 2 (SARS-CoV-2), also known as coronavirus disease 2019 (COVID-19), spreads worldwide [1–3]. New mutant strains of SARS-CoV-2 keep emerging, challenging the wisdom of scientists^2^ and posing a severe threat to human health. SARS-CoV-2 is a highly pathogenic coronavirus that can cause zoonotic diseases. According to the current studies, coronavirus stinger proteins bind specifically to cellular receptors (angiotensin-converting enzyme 2, ACE2; dipeptidyl peptidase 4, DPP4), which mediates viral entry into the cell. Coronaviruses have a potent genomic RNA that directs the synthesis of viral nucleic acids and proteins assembled into new coronaviruses and secreted extracellularly [4]. And viral particles can also bind to host factors, cell surface serine protease TMPRSS2, to promote viral endocytosis and fusion at the cell membrane [5]. Gene sequencing results show that TMPRSS2 can coexpress ACE2 on several tissues, such as nasal mucosa, lung epithelium, and bronchial epithelial tissue, which explains the preference for coronavirus infection in the respiratory system [6, 7]. In addition, accessory proteins on coronaviruses, associated with viral variability, show limited conservation [8]. Studies have shown that additional proteins are related to viral infection immunity. Unfortunately, the function and properties of accessory proteins are still largely unknown [9].

Chronic obstructive pulmonary disease (COPD) is a group of systemic diseases in which the airways are severely involved [10], and airway epithelium is damaged, leading to decreased airway barrier function. Immunohistochemical staining of COPD patients who smoke showed significantly higher expression of ACE2 protein than in normal nonsmoking controls [11], which increases the opportunity for SARS-CoV-2 infections. A clinical study from China showed that 62.5% of patients with severe COVID-19 were comorbid COPD and 25% of COVID-19 patients who died had COPD [12]. The high rate of severe disease and increased mortality in patients with COPD and COVID-19 [13] make the management of SARS-CoV-2 infections more problematic. In addition, high expression of ACE2 and DPP4 can be detected in blood and alveolar lavage fluid in patients with COPD and asthma [14]. Increased expression of ACE2, TMPRSS2, and DPP4 is associated with altered functions of transcription factors that regulate mitochondrial function, telomerase, etc. [15]. In the setting of COPD airway barrier dysfunction, SARS-CoV-2 infection leads to severe airway damage to the mucosa. Pathology shows diffuse alveolar damage and severe perivascular T cell infiltration with extensive neovascularization and microthrombosis [16]. The above studies suggest that COPD is a significant risk factor for poor prognosis in SARS-CoV-2 infections [14]. It makes the association between COPD and COVID-19 a hot topic of interest.

Nowadays, a considerable amount of information is used to describe the mechanisms of physiological functions of cells and tissues through different biosequencing. Complex data, practical software programs, and multisystem modeling platforms constitute a data-driven multisystem bioinformatics ecosystem [17]. The use of systems biology allows us to understand the interactions and information flow in different dimensions within cells, tissues, and organisms [18]. During the COVID-19 epidemic, sequencing of the COVI-19 genes combined with available databases and computational tools allows analysis of protein sequences, prediction of vaccine targets, and potential B and T cell targets for immune modeling [19]. The use of bioinformatics during the COVID-19 epidemic has significantly deepened the study of the virus and shortened the development cycle of treatments and vaccines against the virus. Our study seeks to find bioinformatics links between COPD and COVID-19. First, we analyze the COVID-19 dataset GSE147507 and COPD dataset GSE106986 to look for common differentially expressed genes (DEGs). And we explore the Gene Ontology (GO) and Kyoto Encyclopedia of Genes and Genomes (KEGG) regulatory networks and then analyze the hub genes, which are the core of the gene regulatory network. We search for the regulation with transcription factors (TFs), miRNAs, and targeted drug candidates based on common DEGs. The use of bioinformatics during the COVID-19 epidemic has significantly deepened the study of the virus and shortened the development cycle of treatments and vaccines against the virus [20].

## 2. Materials and Methods

### 2.1. Dataset Collection in This Study

COPD is a susceptibility factor for the severe acute respiratory syndrome coronavirus 2 (SARS-CoV-2) infection. We collected data from the National Center for Biotechnology Information (NCBI) (https://www.ncbi.nlm.nih.gov/geo/) GEO database on microarray, and RNA-seq expression profiling by high-throughput sequencing of SARS-CoV-2 infection in human lung epithelial and alveolar cells was performed on the GSE147507 dataset. The GSE147507 dataset was provided by the GPL18573 Illumina NextSeq 500 expression platform. 19 samples were collected in the GSE106986 dataset, including 5 on normal nonsmoking controls and 14 on COPD groups assay results. We performed a microarray-based analysis of GSE106986 and GSE147507.

### 2.2. Identification of DEGs among COVID-19 and COPD

The DEGs were the basis for our study. The GSE147507 dataset was obtained using the DESeq2 method with R (version 3.6.3) software, and a cutoff criterion of *P* value < 0.05 and log2-fold change (absolute) > 1.0 was chosen for normal controls, and SARS-CoV-2 infected differential genes. GSE106986 was obtained through the GEO2R (https://www.ncbi.nlm.nih.gov/geo/geo2r/) web tool, which uses the limma package for identifying DEGs of COPD and control individuals. Cutoff values for the database were taken as *P* value < 0.05 and log2-fold change (absolute) > 1.5. The expression of common DEGs to both datasets was obtained through the online data analysis library Sangerbox (http://sangerbox.com/).

### 2.3. GO and KEGG Enrichment Analyses of Common DEGs

The GO term analysis of genes includes biological processes, molecular function, and cellular components. KEGG pathways describe signaling pathways associated with disease metabolism and are often annotated with signals. GO and KEGG enrichment analysis data for common genes are available through the (http://enrich.shbio.com/) online database, and analysis maps are available from the online analysis website (http://www.bioinformatics.com.cn/).

### 2.4. The PPI Network Analysis

PPIs are active and passive processes that reflect protein interactions in cellular tissues. The execution of protein functions requires different protein interactions to be realized. We used STRING (https://string-db.org/) database to construct a network of protein interactions and Cytoscape (V.3.6.0) software to implement network mapping to describe common protein's biological and physical roles interactions in COVID-19 and COPD. Cytoscape (V.3.6.0) is an open-source network visualization platform that is a flexible tool to integrate multiple datasets and improve the performance of different interactions, such as PPIs, gene interactions, protein-DNA interactions, and others [21].

### 2.5. Hub Gene Extraction

PPI network consists of node, edge, and the connection between them, among which hub gene is considered the core of the PPI network. We use the Cytohubba plugin in Cytoscape (V.3.6.0) software to extract the hub gene. And we use the degree algorithm to realize the analysis of learning genes. Based on the principle of the clustering coefficient of the Cytohubba plugin, the top 5 hub genes are used to estimate the shortest available path.

### 2.6. Recognition of TFs and miRNAs Engages with Common DEGs

TFs are specific proteins bound to target genes responsible for the rate and information of transcription of genetic information and are essential for controlling genetic information [22]. We analyze DEGs associated TFs through the online database NetworkAnalyst (https://www.networkanalyst.ca/). NetworkAnalyst is a comprehensive data platform that can analyze gene regulatory networks, gene coexpression networks, and pharmacogenomics networks [23]. We implemented transcription factors analysis of differential gene regulation through the JASPAR database network. In addition, we used the TF-miRNA coregulatory interaction database to analyze how miRNAs interact with DEGs. The miRNA interaction information is obtained from the RegNetwork repository. We used the TF-miRNA database to detect miRNAs' biological functions and characteristics interacting with common DEGs.

### 2.7. Protein-Drug Interaction Analysis

Protein-drug interaction analysis data is analyzed from the DSigDB database on the Enrichr (https://maayanlab.cloud/Enrichr/) platform. We obtain drug information to predict potential drugs for protein targets on the DrugBank database (https://go.drugbank.com/).

### 2.8. Molecular Docking

The candidate drugs' chemical crystals were obtained from the PubChem database (https://pubchem.ncbi.nlm.nih.gov/), and we converted the molecular structure to the mol2 format using OpenBabel software. The top 5 hub gene molecular forms were obtained from the Uniport database (https://www.uniprot.org/) and were removed from the corresponding ligands and water molecules by PyMOL software. The molecular docking analysis of compounds and target genes was obtained from the Swissdock database (http://www.swissdock.ch/). The obtained results can be used to interpret the predictions of protein molecule binding.

## 3. Results

### 3.1. The Common DEGs of COVID-19 and COPD

**Figure 1:**
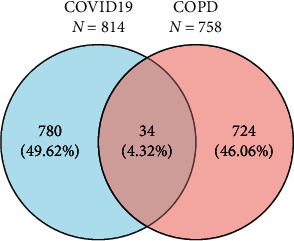
Coexpression analysis of the GSE147507 datasets for COVID-19 and GSE106986 datasets for COPD with differential genes. The cross-tabulation study showed 34 common DGEs.

The common DEGs were acquired to study the changes in gene expression for patients comorbid COVID-19 and COPD. The intercept value condition (*P* value < 0.05 and log2-fold change (absolute) > 1.0) was met for 814 differential genes in the COVID-19 dataset, and 758 differential genes were identified in the COPD dataset. By different statistical methods, we obtained 34 common DEGs (Figure 1). These common DGEs were used to analyze further experimental data.

### 3.2. Gene GO and KEGG Enrichment Analyses

**Figure 2:**
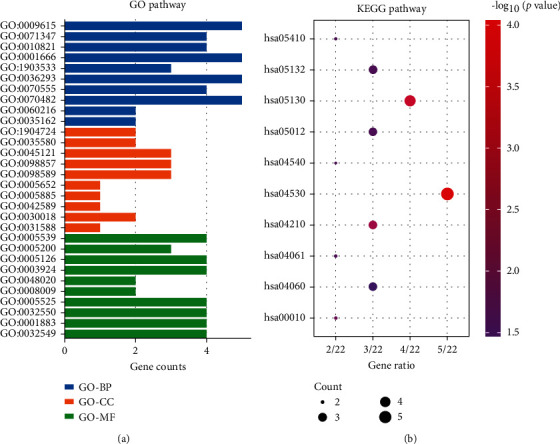
The GO and KEGG enrichment analyses. (a) The BP-, CC-, and MF-related GO terms of 34 common DEG identification results. (b) The top 10 terms of KEGG analysis result of 34 common DEGs. The redder the bubble, the more genes are enriched.

GO and KEGG pathway enrichment analyses for common DGEs is obtained using online databases. The GO terms include biological process (BP), molecular functions (MF), and cellular components (CC). We analyzed the top 10 GO terms (Table 1 and Figure 2(a)). Our results indicate that the BP subgroup is highly concentrated on neutrophil chemotaxis, chemokine-mediated signaling pathway, and lymphocyte chemotaxis. The MF mainly focuses on glycosaminoglycan binding, structural constituent of cytokines, and chemokine receptor binding. The CC especially involves tertiary granule lumen, tertiary granule, and nuclear-activated protein kinase complex. The analysis of KEGG signaling pathways is mainly enriched in viral protein interacting with cytokine and cytokine receptor, tight junction, and cytokine-cytokine receptor (Table 2 and Figure 2(b)).

### 3.3. The PPI Network Analysis of Common DEGs and the Extraction of the Hub Genes

**Figure 3:**
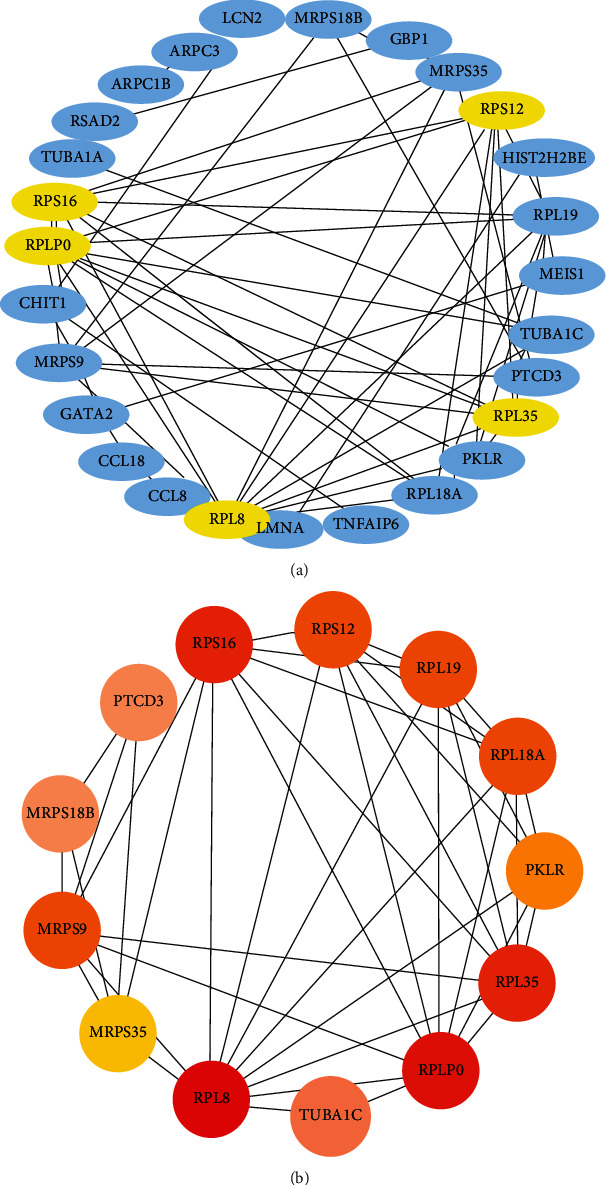
The PPI network for common DEGs is shared by COVID-19 and COPD. (a) The interaction network of 34 common DEGs. These five genes are considered hub genes according to their degree value. (b) The PPI network of top 10 hub genes with neighbors. The redder the color, the tighter the gene connectivity, and the edges indicate the connectivity between two nodes.

The common DEGs were provided to STRING online database to obtain the interaction network of coexpressed genes. The data obtained from STRING are provided to Cytoscape software for further network mapping. The protein-protein interaction (PPI) network contains 27 nodes and 218 edges, and these results are shown in Figure 3(a).

The extraction of the hub genes is achieved using the Cytohubba plugin in Cytoscape software. We find that the first five hub genes are RPL8, RPLP0, RPL35, RPS16, and RPS12. The association of the hub genes with neighbors in the PPI network is shown in Figure 3(b). The hub genes network includes 13 nodes and 156 edges. The redder color indicates the higher connection tightness. Topological analysis for the hub genes (RPL8, RPLP0, RPL35, RPS16, and RPS12) is identified using Cytohubba. The topological analysis result is presented in Table 3. At the same time, our analysis in the Enrichr database (https://maayanlab.cloud/Enrichr/) shows that these hub genes are associated with heart disease, tuberculosis, asthma, anemia, etc. (Table 4).

### 3.4. TFs or miRNA and Gene Interactions

**Figure 4:**
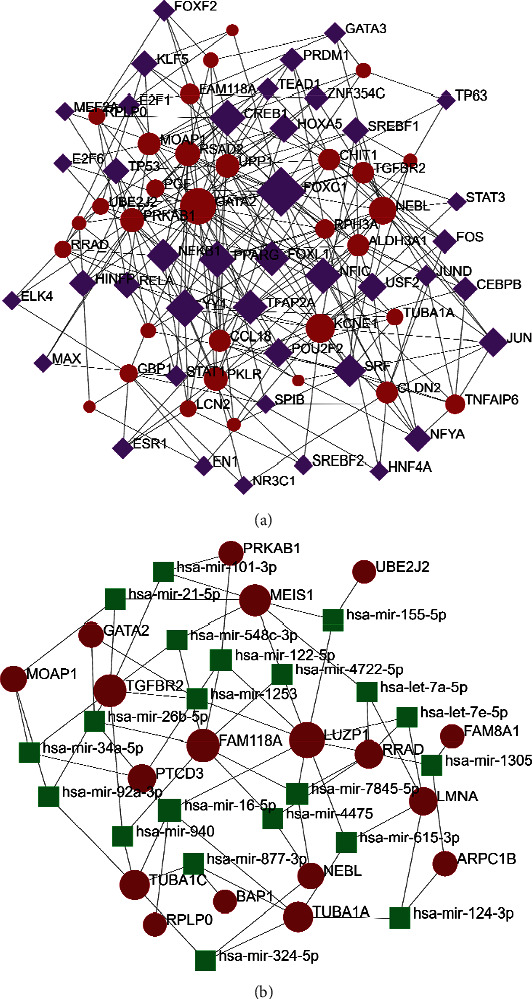
TF or miRNA interactions with common DEGs. (a) TF interaction with common DEGs. (b) miRNA interaction with the common DEGs network analysis. Red represents common DEGs, purple represents TF, and the green represents miRNA.

NetworkAnalyst implemented TFs and common DEG interactions. The results of TF-gene interactions are shown in Figure 4(a). The 34 common DEGs related to transcription factors were selected—genes with at least 3 nodes, resulting in the selection of 32 DEGs. We can see that a target gene is regulated by more than one transcription factor, and one transcription factor can regulate multiple target genes. We also analyzed the miRNAs involved in the regulation of the target genes. The results showed 18 target genes, 39 nodes, and 67 edges in the network (Figure 4(b)).

### 3.5. TFs and miRNA Coregulatory Interactions

**Figure 5:**
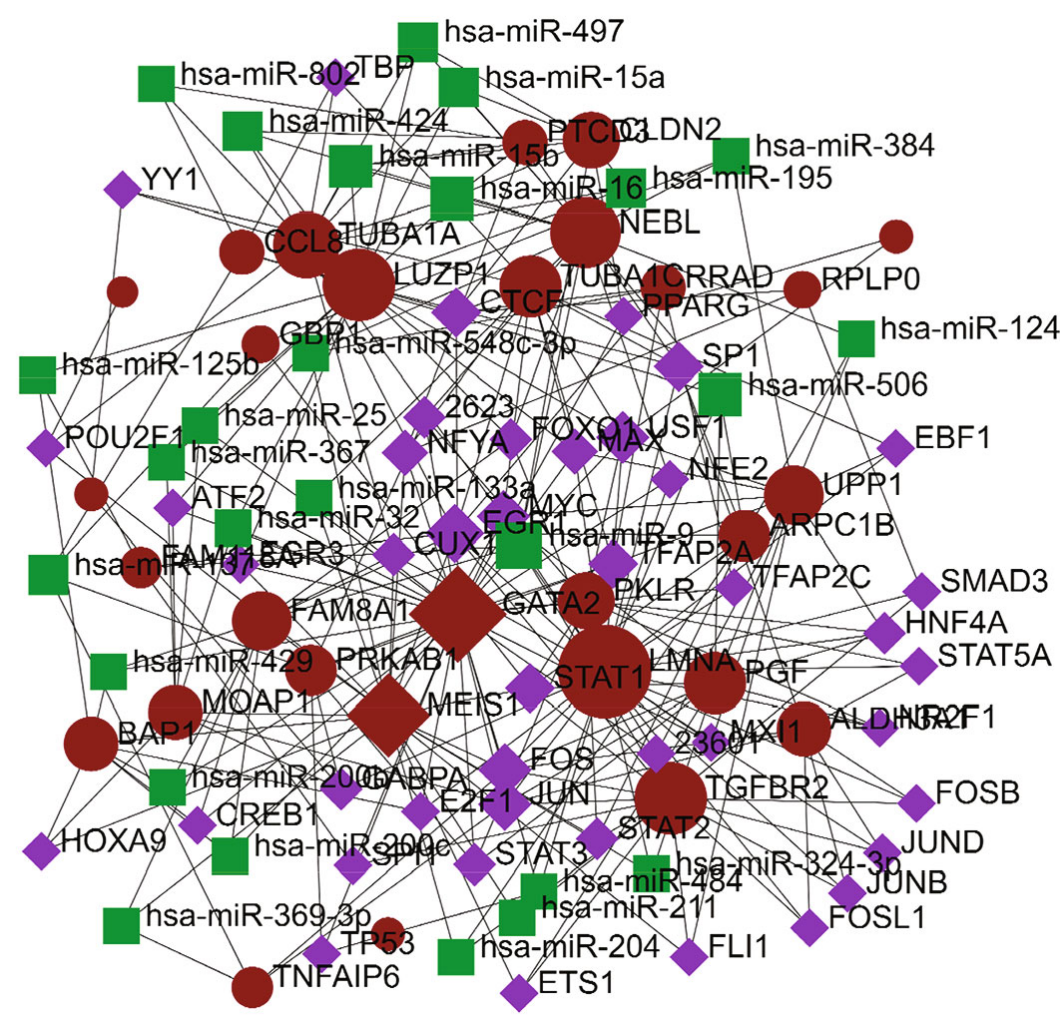
TF-miRNA network regulating common DEGs. The network contains 29 common DEGs, 43 TFs, and several miRNAs involved in regulation. Red represents common DEGs, purple represents TF, and the green represents miRNA.

TFs and miRNAs interaction network was extracted by NetworkAnalyst. TF-miRNA regulatory network is analyzed based on common DEGs. We obtained 29 common DEGs by screening genes with at least 3 nodes. We got the associated TFs and miRNA network, and the regulatory network is shown in Figure 5. It can be seen that there are 98 nodes in the network and 271 edges. 43 TFs and 26 miRNAs are involved in regulating common DEGs.

### 3.6. The Selection of Genetic Candidate Drugs

Drug acquisition is the ultimate aim of disease research. We analyzed the common DEGs in COPD and COVID-19, and the results showed that 5 of the hub genes were selected. After studying the Enrichr platform DSigDB database, we obtained the possible drug candidates (Table 5). The binding of the drug candidate to the target protein was then further evaluated by molecular docking, and the higher the molecular docking score, the more stable the result and the closer to the natural conformation of the compound (Table 6). The results showed that dexverapamil MCF7 UP, STOCK1N-35696 PC3 DOWN, and artesunate CTD 00001840 were considered the optimal drugs to target common DEGs.

## 4. Discussion

COPD is a risk factor for mortality rates in patients with COVID-19. Decreased airway mucosal barrier function is an essential physiological basis for SARS-CoV-2 susceptibilities. Our results explain from a bioinformatic approach that COPD shares a common genetic origin with COVID-19. We analyzed differential gene expression from two gene RNA-seq datasets of COPD and COVID-19. The 34 common DGEs are used to analyze disease-related signaling pathways and network regulatory relationships, which helps find potential biological targets and drug candidates for both diseases.

The GO enrichment analysis is the basis of bioinformatics analysis. It is mainly reflected in the biological process (molecular activities), molecular function (activities of molecular level), and cellular component [24, 25]. The GO analysis of 34 common DGEs explains the role of enriched genes [26]. In terms of the biological process, 34 common DEGs are mainly enriched in response to the virus and cellular response to interleukin-1. SARS-CoV-2 infections can be followed by a severe inflammatory waterfall-like response, causing massive inflammatory cell activation and releasing large amounts of inflammatory factors such as IL-1, IL-6, and IL-18 [27–29]. High-dose interleukin-1 blockade can cause a decrease in serum C-reactive protein and improve respiratory function in COVID-19 patients around 21 days [30]. In addition, IL-1*β*, as a more potent proinflammatory factor, can promote the release of IL-8 and IL-6 and the increase of lymphocytes and dendritic cells in lung tissue during the inflammatory trigger phase [31–34]. This effect may be one of the driving factors of cytokine release syndrome and systemic inflammatory response syndrome after SARS-CoV-2 infection [28, 35]. The common DEG regulating functions are mainly enriched in tertiary and specific granule lumen. They may be associated with virus replication in the host, similar to Taz et al. [36]. For describing the enrichment of molecular function in terms of activities at the molecular level, these 34 common DEGs mainly focus on chemokine receptor binding and glycosaminoglycan binding. Elevated levels of chemokines promote leukocyte aggregation in lung tissue and immune responses and exacerbate inflammatory injury [37]. High-throughput and ultrasensitive proteomics platforms showed that the serum of cytokines and chemokines such as CCL8 and CXCL10 in COVID-19 patients was significantly higher than in healthy controls [38]. Similar findings were observed in animal experiments, where chemokines increased in 3 days after viral infection, and levels of chemokines such as CCL8 and CCL2 continued to expand after 7 days despite a decrease in viral load [39]. Our analysis suggests that CCL8, CCL18, and other chemokine receptor binding are enriched in the gene expression profile of patients who suffered COVID-19 and COPD, which may contribute to the high rate of severe illness and mortality. In the KEGG enrichment analysis, common DEGs were mainly enriched in signaling pathways such as tight junction and viral protein interaction with cytokine and cytokine receptors. Cytokine storms are related to the severity of COVID-19 and serve as an essential risk factor for COVID-19 death [40]. The chemotactic monocytes produce high levels of chemokines and cytokines and induce delayed release of IFN-*α*/*β* after SARS-CoV-2 infection, resulting in increased apoptosis of epithelial and endothelial cells, alveolar edema, and vascular infiltration, which in turn cause respiratory failure [40–42]. This suggests that excessive release of cytokines is a major factor in the expansion of inflammation during COVID-19. An observational study showed that 35 in 37 patients with severe pneumonia developed acute respiratory distress syndrome and required mechanical ventilation when they came with COVID-19 [43].

Many TFs have been associated with COPD pathogenesis in current studies, such as NFKB1, E2F1, YY1, KLF5, FOS, and HNF4A TP63 [26, 44–50]. These TFs are related to COPD, especially its acute exacerbations. TFs regulated mucus secretion in COPD patients, such as HOXA5, activated the Notch signaling pathway, inhibited cupped cell differentiation, and produced excess airway mucus [51]. A recent study about the genomics of COVID-19 showed that TFs like FOXC1, GATA2, YY1, FOXL1, and NFKB1 regulated the inflammatory network in SARS-CoV-2 infections [52]. It suggests more common TFs in both COVID-19 and COPD. We speculate that this may contribute to the medical development of COVID-19 in COPD patients. In particular, specific TFs are significantly associated with receptor ACE2 in SARS-CoV-2-infected hosts, such as GATA2 and HNF4A, promoting or repressing the expression of ACE2 [53]. In addition, we found that TFs coregulate COVID-19 with miRNAs [54, 55]. In our target genes, hsa-miR-200 regulates target genes like BAP1, TNFAIP1, and TF HOXA9. At the same time, data analysis showed that hsa-miR-200 could also affect ACE2/TMPRSS2 expression [56]. TFs and miRNAs jointly regulate target genes and are involved in the development and progression of COPD and COVID-19 comorbidity.

Much current research on SARS-CoV-2 infection drugs is still at the clinical trial stage. The effectiveness of currently used antibodies against viral invasion, nucleotide analogs against viral replication, and protease inhibitors against viral particle formation for SARS-CoV-2 infections still deserves further improvement [57]. Our bioinformatic data analysis shows that artesunate CTD 00001840, dexverapamil MCF7 UP, and STOCK1N-35696 PC3 DOWN may be helpful in the treatment of COVID-19. Artesunate was an antimalarial drug for its prominent role in inhibiting proliferation and inflammation and promoting apoptosis [58]. Studies have shown that artesunate and its derivatives inhibit SARS-CoV-2 activity in vitro, which show concentrations 10-160 times higher in lung tissue than in blood [59, 60]. A domestic observational clinical study showed that for patients treated with artesunate, the time to clinical improvement, the time to SARS-CoV-2 conversions, and the days in the hospital were significantly shorter than the control group [61]. On a mouse model of cigarette smoke-mediated COPD, investigators found that artesunate alleviated cigarette smoke-mediated airway remodeling [62]. The above studies suggest that artesunate treatment may benefit patients with COPD and COVID-19. ACE2 is a receptor of SARS-CoV-2, so angiotensin-converting enzyme inhibitors and angiotensin receptor blockers may upregulate the ACE2 levels in hypertensive patients with COVID-19. Alternatives to this kind of drug need to be sought. Investigators believe that dexverapamil is a better substitute, and animal experiments have shown that dexverapamil does not affect ACE2 expression [63]. STOCK1N-35696 PC3 DOWN is a drug for anti-HIV treatment. It has been demonstrated that STOCK1N-35696 PC3 DOWN can inhibit SARS-CoV-2 infection of cells by binding to cell membrane ACE2 in cellular assays [64]. Gabexate PC3 UP is used chiefly to treat acute pancreatitis [65, 66] and is a serine protease inhibitor [67]. Studies have shown that gabexate inhibits the inflammatory expression of IL-6 and IL-10 and attenuates the inflammatory effects of influenza pneumonia [68]. In any case, these findings will give us therapeutic direction for COVID-19 in clinical practice.

This study integrates the bioinformatics data of COVID-19 and COPD, which is expected to give potential therapeutic agents for COVID-19 treatment. We will provide more data to support these potential drugs in future clinical and experimental trials.

## Figures and Tables

**Table 1:** The GO pathways and their corresponding *P* value and description for common DEGs.

GO	ID	Description	Gene ID	*P* value
BP	GO:0009615	Response to virus	DMBT1/GBP1/LCN2/CCL8/RSAD2	1.84E-04
	GO:0071347	Cellular response to interleukin-1	GBP1/LCN2/CCL8/CCL18	2.10E-04
	GO:0010821	Regulation of mitochondrion organization	LMNA/BAP1/MOAP1/UBE2J2	2.24E-04
	GO:0001666	Response to hypoxia	ALDH3A1/LMNA/PGF/PKLR/TGFBR2	2.88E-04
	GO:1903533	Regulation of protein targeting	KCNE1/BAP1/UBE2J2	3.24E-04
	GO:0036293	Response to decreased oxygen levels	ALDH3A1/LMNA/PGF/PKLR/TGFBR2	3.31E-04
	GO:0070555	Response to interleukin-1	GBP1/LCN2/CCL8/CCL18	3.65E-04
	GO:0070482	Response to oxygen levels	ALDH3A1/LMNA/PGF/PKLR/TGFBR2	4.41E-04
	GO:0035162	Embryonic hemopoiesis	GATA2/TGFBR2	5.49E-04
	GO:0060216	Definitive hemopoiesis	GATA2/MEIS1	4.98E-04

CC	GO:1904724	Tertiary granule lumen	CHIT1/TNFAIP6	3.82E-03
	GO:0035580	Specific granule lumen	CHIT1/LCN2	4.82E-03
	GO:0045121	Membrane raft	KCNE1/TGFBR2/TUBA1A	1.55E-02
	GO:0098857	Membrane microdomain	KCNE1/TGFBR2/TUBA1A	1.56E-02
	GO:0098589	Membrane region	KCNE1/TGFBR2/TUBA1A	1.72E-02
	GO:0005652	Nuclear lamina	LMNA	1.83E-02
	GO:0005885	Arp2/3 protein complex	ARPC1B	1.83E-02
	GO:0042589	Zymogen granule membrane	DMBT1	1.83E-02
	GO:0030018	Z disc	KCNE1/NEBL	2.05E-02
	GO:0031588	Nucleotide-activated protein kinase complex	PRKAB1	2.32E-02

MF	GO:0008009	Chemokine activity	CCL8/CCL18	3.32E-03
	GO:0005539	Glycosaminoglycan binding	PGF/CCL8/TGFBR2/TNFAIP6	6.53E-04
	GO:0005200	Structural constituent of cytoskeleton	TUBA1A/ARPC1B/TUBA1C	7.43E-04
	GO:0048020	CCR chemokine receptor binding	CCL8/CCL18	2.56E-03
	GO:0005126	Cytokine receptor binding	PGF/CCL8/CCL18/TGFBR2	1.49E-03
	GO:0003924	GTPase activity	GBP1/RRAD/TUBA1A/TUBA1C	2.35E-03
	GO:0005525	GTP binding	GBP1/RRAD/TUBA1A/TUBA1C	3.93E-03
	GO:0032550	Purine ribonucleoside binding	GBP1/RRAD/TUBA1A/TUBA1C	4.09E-03
	GO:0001883	Purine nucleoside binding	GBP1/RRAD/TUBA1A/TUBA1C	4.20E-03
	GO:0032549	Ribonucleoside binding	GBP1/RRAD/TUBA1A/TUBA1C	4.24E-03

**Table 2:** The KEGG pathways and their corresponding *P* value and description for common DEGs.

ID	Description	*P* value	Gene ID
hsa04530	Tight junction	7.46E-05	PRKAB1/TUBA1A/CLDN2/ARPC1B/TUBA1C
hsa04061	Viral protein interaction with cytokine and cytokine receptor	2.99E-02	CCL8/CCL18
hsa04060	Cytokine-cytokine receptor interaction	4.45E-02	CCL8/CCL18/TGFBR2
hsa05130	Pathogenic Escherichia coli infection	1.78E-03	TUBA1A/CLDN2/ARPC1B/TUBA1C
hsa04210	Apoptosis	5.69E-03	LMNA/TUBA1A/TUBA1C
hsa00010	Glycolysis/gluconeogenesis	1.41E-02	ALDH3A1/PKLR
hsa04540	Gap junction	2.35E-02	TUBA1A/TUBA1C
hsa05410	Hypertrophic cardiomyopathy	2.46E-02	LMNA/PRKAB1
hsa05012	Parkinson disease	2.89E-02	TUBA1A/TUBA1C/UBE2J2
hsa05132	Salmonella infection	2.89E-02	TUBA1A/ARPC1B/TUBA1C

**Table 3:** Topological result exploration for top five hub genes.

Hub gene	Degree	Stress	Closeness	Betweenness	Eccentricity	Clustering coefficient
RPL8	10	102	11.5	38.17	0.26	0.58
RPLP0	9	62	11	22.17	0.26	0.67
RPL35	8	26	10.33	5.5	0.17	0.82
RPS16	8	40	10.33	9.833	0.17	0.71
RPS12	7	2	9.5	0.33	0.17	0.95

**Table 4:** The enrichment relationship between core genes and diseases.

Disease term	*P* value	Combined score
Aase-Smith syndrome	5.00E-03	1.39E+03
Anemia, macrocytic	2.48E-02	5.02E+01
ECG abnormality	1.17E-02	1.08E+02
Tuberculosis	2.55E-02	4.88E+01
Asthma	3.26E-02	1.30E+02
Non-small-cell lung carcinoma	4.75E-02	1.79E+02
Secondary malignant neoplasm of liver	5.00E-03	1.39E+03

**Table 5:** Candidate drug compounds for COVID-19 and COPD.

Name of drug	DrugBank number	Chemical formula	*P* value	Genes
Artesunate CTD 00001840	DB09274	C19H28O8	0.011	RPS12
Dexverapamil MCF7 UP	DB14063	C27H38N2O4	0.020	PKLR
STOCK1N-35696 PC3 DOWN	DB00625	C14H9ClF3NO2	0.018	RPL18A
hydralazine CTD 00006108	DB01275	C8H8N4	0.020	PL18A/RPS12
Gabexate PC3 UP	DB12831	C16H23N3O4	0.020	PKLR
Disodium selenite CTD 00007229	DB11127	Na2O3Se	0.003	RPS16/RPL18A/RPLP0/RPL8
FERRIC AMMONIUM CITRATE CTD 00000709	DB09501	C6H11FeNO7	0.009	MRPS9

**Table 6:** Molecular docking analysis of candidate drugs and target genes.

Name of drug	Genes	Estimated affinity (kcal/mol)
Artesunate CTD 00001840	RPS12	-7.44
Dexverapamil MCF7 UP	PKLR	-7.85
STOCK1N-35696 PC3 DOWN	RPL18A	-7.36
Hydralazine CTD 00006108	PL18A	-5.87
	RPS12	-6.59
Gabexate PC3 UP	PKLR	-7.30
Disodium selenite CTD 00007229	RPS16	-3.37
	RPL18A	-4.48
	RPLP0	-2.84
	RPL8	-3.68
FERRIC AMMONIUM CITRATE CTD 00000709	MRPS9	-4.74

## Data Availability

The genes described in detail here were deposited in NCBI.
